# Absolute rather than relative income is a better socioeconomic predictor of chronic obstructive pulmonary disease in Swedish adults

**DOI:** 10.1186/s12939-017-0566-2

**Published:** 2017-05-04

**Authors:** Sten Axelsson Fisk, Juan Merlo

**Affiliations:** Unit for Social Epidemiology, Faculty of Medicine, Lund University, CRC, Jan Waldeströms gata, 35, S-205 02 Malmö, Sweden

**Keywords:** Health inequality - absolute income - relative income, Chronic obstructive pulmonary disease, Materialistic theory, Psychosocial theory, Health equity

## Abstract

**Background:**

While psychosocial theory claims that socioeconomic status (SES), acting through social comparisons, has an important influence on susceptibility to disease, materialistic theory says that socioeconomic position (SEP) and related access to material resources matter more. However, the relative role of SEP versus SES in chronic obstructive pulmonary disease (COPD) risk has still not been examined.

**Method:**

We investigated the association between SES/SEP and COPD risk among 667 094 older adults, aged 55 to 60, residing in Sweden between 2006 and 2011. Absolute income in five groups by population quintiles depicted SEP and relative income expressed as quintile groups *within* each absolute income group represented SES. We performed sex-stratified logistic regression models to estimate odds ratios and the area under the receiver operator curve (AUC) to compare the discriminatory accuracy of SES and SEP in relation to COPD.

**Results:**

Even though both absolute (SEP) and relative income (SES) were associated with COPD risk, only absolute income (SEP) presented a clear gradient, so the poorest had a three-fold higher COPD risk than the richest individuals. While the AUC for a model including only age was 0.54 and 0.55 when including relative income (SES), it increased to 0.65 when accounting for absolute income (SEP). SEP rather than SES demonstrated a consistent association with COPD.

**Conclusions:**

Our study supports the materialistic theory. Access to material resources seems more relevant to COPD risk than the consequences of low relative income.

## Background

By 2020, chronic obstructive pulmonary disease (COPD) is predicted to become the fifth largest disease burden and the third cause of death globally [1, 2]. The Swedish National board of Health and Welfare estimates that about 500 000 people in Sweden suffer from COPD, but only approximately 100 000 of them have established diagnoses [3].

Smoking is the major risk factor for developing COPD [4] and is more frequent among people with socioeconomic disadvantage, which is consistent with the higher prevalence of COPD in that group [5–7]. Socioeconomic differences in COPD risk remains when controlling for smoking [6], so other independent mechanisms could explain those differences [8–12]. An open discussion in social epidemiology concerns the relative importance of material versus psychosocial factors in the genesis of socioeconomic differences in health [13–15].

Socioeconomic position (SEP) is often operationalized by using information on absolute income. Materialistic theory assumes that an individual’s health depends on their own (and only their own) level of income, rather than that of those around them. It is a person’s SEP and the related access to material resources and power that matters most [9, 16]. Although this approach to social class has been criticized by Marxist health researchers for transforming a societal process into an individual characteristic, it can be considered a pragmatic class definition [17]. For instance, deprivation in low household SEP might impair intra-uterine and childhood environments [18], increasing the risk of growth restriction and of repeated viral infections. In turn, decreased pulmonary reserve capacity might then predispose an individual to COPD later in life [19]. Sociological mechanisms in adulthood may promote smoking habits [20], low physical activity, and inappropriate nutrition [21] in people with low SEP. These factors alone or in combination with harmful occupational exposures [22], air pollution [23, 24], and reduced access to appropriate health care and medication may also increase risk of developing COPD in people with low SEP [2].

In contrast, the psychosocial theory focuses on characteristics such as low social cohesion, income inequalities and the experience of relative poverty in the understanding of mechanisms behind the social health gradient. In this study we investigate whether low income compared to people in the same strata of society is related to incidence of COPD. One key question is the individual’s socioeconomic status (SES), which can be operationalized by using information on relative income, in relation to the reference socioeconomic group. In this view, those with relatively lower income within a high SEP group will show a higher COPD risk even if access to material resources is high for the entire reference group. The harmful effect is hypothesized to act by mechanisms precipitated by psychosocial harm (e.g., shame, loss of self-respect) from social comparisons [16, 25] directly related to the individual’s SES [16]. The psychosocial model emphasizes that relative income inequalities are relevant not only to the poor, but also to the middle and even upper strata [25–27]. Low SES is assumed to cause stress that activates neuroendocrine systems, especially the sympathetic response and the hypothalamus-pituitary-adrenal-axis (HPA-axis). Chronic psychological stress increases cortisol levels via the HPA-axis [28], which has harmful effects when the stress response is prolonged [29, 30]. Low relative income could cause COPD through lowered immunity due to increased cortisol levels and predisposition to infections. Chronic stress is also detrimental through promoting inappropriate coping behaviours, such as excessive alcohol consumption, smoking, and unhealthy eating [31]. So far, the role of psychosocial stress in COPD seems not to have been as thoroughly studied as it has in cardiovascular diseases.

It is difficult, not to say impossible, to isolate the relative and the absolute income hypotheses, especially when it comes to identifying the appropriate reference groups for social comparison [9]. There is also an underlying political tension. One criticism of psychosocial explanation models is their propensity to “blame the victim”. If psychosocial stress is due to low status and lack of supportive relationships in deprived neighbourhoods, could we not simply teach the poor to be less stressed? [16]. Some authors [13], but not others [26], claim that exaggerated focus on material conditions might misdirect policies.

Only a few investigations [32–34] have examined the socioeconomic differences in COPD in Sweden and, as far we know, no one in the global literature has assessed the relative relevance of SEP versus SES to incidence of COPD. Therefore, we aimed to analyse those questions using a nationwide cohort of adults aged 55 to 60 and residing in Sweden in 2011.

## Methods

### Study population

The National Board of Health and Welfare, in coordination with Statistics Sweden, linked the register of the Total Swedish Population to other national databases such as the National Inpatient Register, the National Mortality Register, and the Income and Asset Register. This record linkage was performed by the Swedish authorities using a unique personal identification number given to each person residing in Sweden. In the data we analysed, the identification numbers were replaced with arbitrary numbers to safeguard the anonymity of the subjects.

**Fig. 1:**
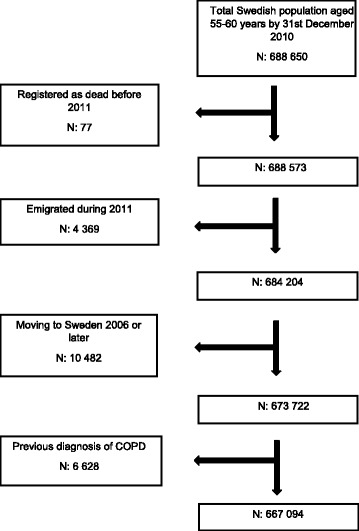
Study sample

The process of selection of individuals included in the study database is visualized in Fig. 1. From the initial 688 650 individuals aged 55 to 60 years and residing in Sweden by the baseline date of December 31st, 2010, we excluded 77 who died before 2011 and were erroneously registered in the population file. Since age is associated to both income level and COPD risk, we restricted our study by selecting a narrow age span (i.e., 55-60) in order to reduce the confounding influence of age. To ensure information on incident COPD during 2011 we excluded 4 369 individuals who emigrated during 2011. 10 482 individuals residing in Sweden less than four years were also excluded to make sure that information on prevalence of COPD was available. Finally, we excluded individuals with a COPD diagnosis within the four years before baseline, which rendered a final study sample of 667 094 individuals.

### Assessment of variables

The variable COPD was defined as a hospital discharge or visit to a hospital clinic diagnosis with one of the following International Statistical Classification of Diseases and related Health Problems 10^th^ revision [35] (ICD-10) codes: J40 (bronchitis, not specified as acute or chronic), J41 (simple and mucopurulent chronic bronchitis), J42 (unspecified chronic bronchitis), J43 (emphysema), or J44 (other chronic obstructive pulmonary disease). We identified new cases of COPD from January 1st, 2011 to December 31st, 2011.

We calculated household individualized disposable income by dividing the total disposable income of a family by the number of family members, taking into account the different consumption weight of adults and children according to Statistics Sweden. We operationalized SEP by categorizing the income into quintiles of the whole Swedish population between 35 and 80 years old. That is, we created five groups of *absolute income* as the groups were created using the whole population. The five groups were named *high income*, *medium high income*, *medium income*, *medium low income,* and *low income*. To operationalize SES we calculated a relative income variable by making new quintiles within each quintile of absolute income. E.g. all people that belonged to the poorest quintile within their absolute income quintiles were pooled together to compose the *low relative income group.* We used the same category labels as those used for absolute income and the *high income* category as reference in the analyses.

We defined *age* as age in 2010 and *sex* as binary: legally male or female.

### Statistical and epidemiologic methods

We performed sex-stratified logistic regression models to examine the association between COPD risk and age, SEP (absolute income) and SES (relative income). Model A included only the continuous age variable; model B included age and absolute income; model C included age and relative income; and model D included age and both absolute and relative income. We expressed associations by means of ORs and 95% confidence intervals (CI). Since COPD incidence was low, ORs correspond well to relative risks.

We paid special attention to calculating the discriminatory accuracy of the models, as commented in more detail elsewhere [36, 37]. For this purpose, we calculated the area under the receiver-operating characteristic curve (AUC). The AUC measured the ability of the model to correctly classify those with and without COPD assuming a value between 1 and 0.5, where 1 is perfect discrimination and 0.5 is as informative as flipping an unbiased coin. Even though our cohort only included people between 55 and 60 years of age, the incidence of COPD increases with age. Therefore, we calculated the AUC of model A to estimate how much absolute and relative income added in discriminatory accuracy (DA) compared to models using age only.

We used SPSS version 21 (SPSS Inc., Chicago, IL, USA) to perform the statistical analyses.

## Results

Overall, 3.1 per 1000 individuals (1754/667 094) in the study sample suffered a COPD event during 2011. Table 1 shows a clear gradient in the incidence of COPD for absolute income groups, with an absolute risk difference between the *low* and *high income* groups of 3.1 per thousand in both men and women. For relative income groups this risk difference is rather inconsistent in both genders. As expected, the average age was about 57 years in all income groups

**Table 1:** Age and incidence of chronic obstructive pulmonary disease by absolute and relative income groups in the 333 952 men and 333 142 women aged 55 to 60 years and residing in Sweden in 2011

	MEN				WOMEN			
	Age (mean)	Number of cases	Number of people	Incidence (per 1000 individuals)	Age (mean)	Number of cases	Number of people	Incidence (per 1000 individuals)
Absolute income^a^								
Low	57.4	167	38125	4.4	57.4	140	30567	4.6
Medium low	57.4	175	39481	4.4	57.5	208	38624	5.4
Medium	57.4	138	50760	2.7	57.5	182	52913	3.4
Medium high	57.5	161	85677	1.9	57.5	256	95865	2.7
High	57.6	158	119909	1.3	57.6	169	115173	1.5
Relative income^b^								
Low	57.5	150	59477	2.5	57.5	161	58513	2.8
Medium low	57.5	157	62965	2.5	57.5	203	63045	3.2
Medium	57.5	173	67131	2.6	57.5	186	67881	2.7
Medium high	57.5	179	70374	2.5	57.5	221	71018	3.1
High	57.5	140	74005	1.9	57.5	184	72685	2.5

^a^The absolute income is categorized by quintiles of all 4 994 921 people aged 35 to 80 years registered as residents in Sweden by December 31st, 2010. ^b^The relative income categories are defined by quintile groups within absolute income categories

.

Table 2, models 2, 3, and 4 show that both absolute and relative income are associated with COPD risk. In both men and women there is a clear gradient for absolute income groups with increasing COPD risk as the absolute income decreases. However, there is no such gradient for relative income that also shows much smaller ORs.

**Table 2:** Association between absolute and relative income and risk of chronic obstructive pulmonary disease in the 333 952 men and 333 142 women aged 55 to 60 years and residing in Sweden in 2011. Values are OR, 95% CI, and AUC

	Model 1 (Age)	Model 2 (Age and absolute income)	Model 3 (Age and relative income)	Model 4 (Age and absolute and relative income)
	OR (95% CI)	OR (95% CI)	OR (95% CI)	OR (95% CI)
MEN				
Age (1 year)	1.09 (1.05–1.14)	1.05 (1.06–1.15)	1.09 (1.05–1.14)	1.11 (1.06–1.15)
Absolute income^a^				
High		REF		REF
Medium high		1.43 (1.15–1.78)		1.44 (1.16–1.80)
Medium		2.10 (1.67–2.64)		2.11 (1.68–2.65)
Medium low		3.44 (2.77–4.27)		3.44 (2.77–4.27)
Low		3.40 (2.73–4.23)		3.39 (2.73–4.22)
Relative income^b^				
High			REF	REF
Medium high			1.35 (1.08–1.68)	1.34 (1.08–1.68)
Medium			1.37 (1.09–1.71)	1.36 (1.08–1.69)
Medium low			1.32 (1.05–1.66)	1.31 (1.04–1.64)
Low			1.34 (1.06–1.69)	1.31 (1.04–1.64)
AUC (95% CI)	0.54 (0.52–0.56)	0.65 (0.63–0.66)	0.55 (0.53–0.57)	0.65 (0.63–0.67)
WOMEN				
Age (1 year)	1.05 (1.01–1.09)	1.06 (1.02–1.10)	1.05 (1.01–1.09)	1.06 (1.02–1.10)
Absolute income				
High		REF		REF
Medium high		1.83 (1.51–2.22)		1.84 (1.52–2.24)
Medium		2.36 (1.92–2.91)		2.39 (1.94–2.95)
Medium low		3.71 (3.03–4.55)		3.73 (3.05–4.58)
Low		3.17 (2.54–3.97)		3.20 (2.56–4.01)
Relative income				
High			REF	REF
Medium high			1.23 (1.01–1.50)	1.25 (1.03–1.52)
Medium			1.08 (0.88–1.33)	1.11 (0.91–1.36)
Medium low			1.27 (1.04–1.56)	1.34 (1.10–1.64)
Low			1.09 (0.88–1.35)	1.15 (0.93–1.42)
AUC (95% CI)	0.53 (0.51–0.54)	0.63 (0.62–0.65)	0.54 (0.52–0.55)	0.64 (0.62–0.65)

^a^The absolute income is categorized by quintiles of all 4,994,921 people aged 35 to 80 years registered as residents in Sweden by December 31st, 2010. ^b^The relative income categories are defined by quintile groups within absolute income categories

As expected, because of the short age range (55 to 60 years), the AUC for age in model 1 was close to 0.5 in both men and women. Inclusion of absolute income in model 2 increased the AUC to 0.65 in men and to 0.63 in women. Relative income in model 3, on the other hand, did not add much to model 1 (age only), as the AUC was 0.55 in both men and women.

## Discussion

In this large, population-based study both SEP (measured as absolute income) and SES (assessed as relative income) were associated with COPD risk. However, while the association between relative income groups and COPD was rather inconsistent, we found a clear socioeconomic gradient for absolute income groups, which confirms previous findings [5, 6, 38]. COPD risk increased with decreasing absolute income, so ORs for COPD were around three times higher for the poorest than for the richest individuals. Even if the AUC value was rather low for both absolute and relative income, the AUC for absolute income was clearly higher than for relative income. Therefore, our study suggests that the materialistic absolute income model is more relevant than the psychosocial relative income model for understanding socioeconomic disparities in COPD risk. It seems that limited material resources per se (i.e., low SEP) are more relevant to COPD risk than the psychosocial consequences of having relatively less resources than the others with a similar income (i.e., low SES). Similar conclusions have previously been drawn for other health outcomes [14, 39].

By including a measure of DA like the AUC, our study adds a new tool for evaluating the relevance of (socioeconomic) categorizations in public health as recently discussed [37].

### Material or psychosocial mechanisms

Relative income is a complex concept. A fundamental aspect is the difficulty of identifying appropriate reference groups for social comparison. It could be questioned whether individuals compare themselves with people below or above them, or if they compare themselves with others like them or to celebrities and moguls portrayed in the mass media. Kawachi et al. (2002) concluded that most likely people compare themselves simultaneously in several directions.

Our aim was to contribute to the question of whether material or psychosocial mechanisms best explain income-related inequalities in COPD risk. It could be argued that OR and DA for absolute income reflect the effects of psychosocial stress and not of material deprivation. The impaired health observed in the poorest groups could be because poor people compare themselves with the rich people, which leads to chronic stress, higher cortisol levels, and increased general susceptibility to diseases, including COPD. Wilkinson and Picket (2006), for example, argue that it is relative socioeconomic differences between broader groups, such as nations, rather than between neighbourhoods that cause psychosocial stress. Since the psychosocial stress is presumably present across all societal strata, we would have expected a difference in incidence between people with similar absolute incomes but different relative incomes if the incidence of COPD would have depended on the psychosocial comparison. As an alternative to our main analysis including absolute and relative income in the same model, we performed analyses of the association of SES and COPD in separate models within the five strata of absolute income quintiles. However, relative income did not show a consistent gradient within any of the absolute income quintiles.

### Strengths and weaknesses of the study

Our results are derived from a large hospital database comprising the whole Swedish population but we did not have information on COPD diagnoses from primary health care. Hence, we only identified cases treated at the hospital (hospitalizations or visits to an external clinic at the hospital), which may underestimate the incidence of COPD in the population. We do not think this situation had a major influence on our study as our aim was to investigate the contributions of absolute and relative income rather than the exact incidences of COPD in the population. Also, we used ICD codes recorded in routine care rather than in clinical examinations focused on identifying COPD cases in a prospective cohort study. However, hospital ICD codes of COPD from the Swedish Inpatient Registry have been considered to have acceptable validity for epidemiological research in a previous study [40]. If the people that were excluded because of emigration or recent immigration belong to lower SEP-groups and also have a higher risk for COPD our results may underestimate the socioeconomic gradients. We do not believe this affects the conclusions of our study since only 0.7% of the individuals emigrated and 1.6% resided in Sweden less than four years (see section on study population).

The quality of income data is high, including income from wages, subsidies, retirement, insurance, profits on capital, and other sources, according to Statistics Sweden. Lynch and Kaplan [41] suggest that repeated measures of income and assessment of wealth should be included to better reflect life course effects of SEP and total material resources available for an individual. The fact that we only measured income on one occasion can be considered a weakness. For instance, suffering from COPD may lead to a reduced income rather than the opposite. We excluded individuals with previous COPD, which reduces the problem of reverse causality.

When planning the analyses, we considered the existence of common causes of both income and COPD that could confound the association between those variables. Education and occupation are alternative indicators of SEP. If we were to adjust for them, we would underestimate the association between low SEP and COPD. Since prevalence of COPD increases with age [4] and income normally increases with age until retirement, age was the only variable that we adjusted for in addition to choosing a study population with individuals of similar age (i.e., 55 to 60 tears). Different smoking patterns among men and women motivated the sex-stratified analyses [42].

Smoking is the most important risk factor for COPD [4, 23, 43]. It is known to be more prevalent among people with low SEP [44, 45]. Since it is low income that causes smoking rather than smoking that causes low income, adjusting for smoking would underestimate the association between income and COPD.

### Psychosocial versus materialistic interventions

The dichotomous description of psychosocial versus materialistic theories used hitherto is pedagogic but not entirely true. Psychosocial researchers agree that material deprivation exists even in high-income countries and materialistic epidemiologists admit the presence of a psychosocial pathway. Although followers of the materialist theory and those of the psychosocial one disagree on to what extent specific mechanisms explain socioeconomic health gradients, they harmonize about the political direction needed. Effective smoking prevention programmes among low income people would probably reduce the slope of the socioeconomic gradient observed in this study. Solving health problems by teaching the poor to live healthily conveys a risk of blaming the victim if the health problems are the result of the political and cultural system [25]. Therefore, interventions should be directed at up-stream societal causes of those health problems. An equalitarian distribution of resources in the society will lead to better health whether the underlying mechanisms are materialistic, psychosocial, or both. To ameliorate the effects of materialistic inequalities in COPD incidence, investments in public primary health care with greater availability of spirometry could be effective. Subsidized medications, improved housing for children to prevent respiratory infections in early life, and strict regulation of working conditions and air pollution are other materialistic interventions to reduce the social gradient for incidence of COPD. The trend of privatization in primary health care in Sweden has benefited high income groups more than low income groups [46, 47] and therefore may exacerbate the social gradient for COPD incidence.

The psychosocial model of how relative poverty causes bad health is a significant advance over purely behavioural explanations that blame poor people for their unhealthy life styles. Thanks to this, struggles for equality in health have earned broad scientific support. Although suggestions to ameliorate income and class division are presented by psychosocial researchers [25], little attention is directed at the capitalist structure of production as an upstream causes of economic and health gaps in society.

### Future research and conclusion

Our study is innovative as we calculated and interpreted not only measures of association such as the OR but also measures of DA such as the AUC as recently proposed in public health research [37]. By doing so we pioneer a new *imaginative* approach in social epidemiology [48] that goes beyond probabilities to explain heterogeneity around averages [36, 37, 49]. Our study indicates that neither SEP nor SES sufficiently increases the AUC of a model including only age for discriminating patients with, from those without COPD. Therefore, interventions exclusively directed at people with low income might convey the risk of stigmatizing people who already bear a high load of psychosocial stress and impaired material resources. Based on our results, prevention of COPD should not exclusively be understood as a fundamental socioeconomic issue. However, we have used rather simple categorizations of income that may not properly capture the social and economic heterogeneity in the distribution of COPD risk. For instance, the materialistic approach hypothesizes health depends on what resources a person possesses.

This study is based on the assumption that income captures purchasing power. Nevertheless, the same amount of money might be less efficient in “buying health” if, for example, you are a female immigrant and have a low SEP compared to a rich man born in Sweden. We also discussed this in previous studies from the analogous perspective of multilevel analysis of individual heterogeneity [36]. The key idea is to understand social heterogeneity by identifying categories that better discriminate between who suffers from COPD and who not. Future research should include intersectional analyses as a model for identifying socially defined groups that are more vulnerable to poor health outcomes [50]. Given the higher precision when including more variables, it is possible to identify smaller groups suffering from the consequences of structural inequalities and at high risk for COPD and where interventions are more easily affordable than for the whole population. Combining intersectionality theory with measures of discriminatory accuracy may be a useful tool in modern social epidemiology as recently indicated [36, 37].

## Conclusions

In conclusion, it seems that limited material resources per se are more relevant than the psychosocial consequences of having a relatively lower status than others with a similar income. Our results, therefore, suggest that the materialistic explanatory model is more relevant than the psychosocial relative income model for understanding socioeconomic disparities in COPD risk. However, the rather low DA of both SEP and SES suggest that public health interventions should target the structural factors in the whole society, rather than target specific income groups.

## Abbreviations

AUC: Area under receiver-operating characteristic curve
CI: Confidence interval
COPD: Chronic obstructive pulmonary disease
DA: Discriminatory accuracy
OR: Odds ratio
SEP: Socioeconomic position
SES: Socioeconomic status

## References

[1] Agusti AG (2005). COPD, a multicomponent disease: implications for management. Respir Med.

[2] Pauwels RA, Buist AS, Calverley PM, Jenkins CR, Hurd SS (2001). Global strategy for the diagnosis, management, and prevention of chronic obstructive pulmonary disease. NHLBI/WHO global initiative for chronic obstructive lung disease (GOLD) workshop summary. Am J Respir Crit Care Med.

[3] Socialstyrelsen: Nationella riktlinjer - Utvärdering 2014 - Vård vid astma och KOL. vol. 1. pp. 24; 2014:24.

[4] Pierson DJ (2006). Clinical practice guidelines for chronic obstructive pulmonary disease: a review and comparison of current resources. Respir Care.

[5] Gershon AS, Dolmage TE, Stephenson A, Jackson B (2012). Chronic obstructive pulmonary disease and socioeconomic status: a systematic review. COPD.

[6] Kainu A, Rouhos A, Sovijarvi A, Lindqvist A, Sarna S, Lundback B (2013). COPD in Helsinki, Finland: socioeconomic status based on occupation has an important impact on prevalence. Scand J Public Health.

[7] Lange P, Marott JL, Vestbo J, Ingebrigtsen TS, Nordestgaard BG (2014). Socioeconomic status and prognosis of COPD in Denmark. COPD.

[8] Der G (2001). Absolute income and life expectancy. J Epidemiol Community Health.

[9] Kawachi I, Subramanian SV, Almeida-Filho N (2002). A glossary for health inequalities. J Epidemiol Community Health.

[10] Marmot M (2007). Status Syndrome.

[11] Muntaner C, Lynch J, Oates GL (1999). The social class determinants of income inequality and social cohesion. Int J Health Serv.

[12] Muntaner C, Rai N, Ng E, Chung H (2012). Social class, politics, and the spirit level: Why income inequality remains unexplained and unsolved. Int J Health Serv.

[13] Marmot M, Wilkinson RG (2001). Psychosocial and material pathways in the relation between income and health: a response to Lynch et al. BMJ.

[14] Hillemeier MM, Lynch J, Harper S, Raghunathan T, Kaplan GA (2003). Relative or absolute standards for child poverty: a state-level analysis of infant and child mortality. Am J Public Health.

[15] Groffen DA, Bosma H, Tan FE, van den Akker M, Kempen GI, van Eijk JT (2012). Material vs. psychosocial explanations of old-age educational differences in physical and mental functioning. Eur J Public Health.

[16] Lynch JW, Smith GD, Kaplan GA, House JS (2000). Income inequality and mortality: importance to health of individual income, psychosocial environment, or material conditions. BMJ.

[17] Muntaner C, Ng E, Chung H, Prins SJ (2015). Two decades of Neo-marxist class analysis and health inequalities: a critical reconstruction. Soc Theory Health.

[18] Reagan PB, Salsberry PJ, Olsen RJ (2007). Does the measure of economic disadvantage matter? exploring the effect of individual and relative deprivation on intrauterine growth restriction. Soc Sci Med.

[19] From the Global Strategy for the Diagnosis, Management and Prevention of COPD, Global Initiative for Chronic Obstructive Lung Disease (GOLD) 2017. http://goldcopd.org/gold-2017-global-strategy-diagnosis-management-prevention-copd/. Accessed 30 Apr 2017.

[20] Hedman L, Andersson M, Stridsman C, Ronmark E (2015). Evaluation of a tobacco prevention programme among teenagers in Sweden. BMJ Open.

[21] Fernandez-Alvira JM, Bornhorst C, Bammann K, Gwozdz W, Krogh V, Hebestreit A, Barba G, Reisch L, Eiben G, Iglesia I (2015). Prospective associations between socio-economic status and dietary patterns in european children: the identification and prevention of dietary- and lifestyle-induced health effects in children and infants (IDEFICS) study. Br J Nutr.

[22] Wurtz ET, Schlunssen V, Malling TH, Hansen JG, Omland O (2015). Occupational COPD among Danish never-smokers: a population-based study. Occup Environ Med.

[23] Eisner MD, Anthonisen N, Coultas D, Kuenzli N, Perez-Padilla R, Postma D, Romieu I, Silverman EK, Balmes JR (2010). An official american thoracic society public policy statement: novel risk factors and the global burden of chronic obstructive pulmonary disease. Am J Respir Crit Care Med.

[24] Larsson SL, C-G. KOL - en multifaktoriell systemsjukdom. Läkartidningen 2007; 104: 1028-1031. 17476903

[25] Wilkinson RG, Pickett K: The Spirit Level: Why More Equal Societies Almost Always Do Better. Allen Lane; 2009.

[26] Lynch J, Due P, Muntaner C, Smith GD (2000). Social capital--is it a good investment strategy for public health?. J Epidemiol Community Health.

[27] Marmot MG, Smith GD, Stansfeld S, Patel C, North F, Head J, White I, Brunner E, Feeney A (1991). Health inequalities among British civil servants: the Whitehall II study. Lancet.

[28] Tobin M (1997). Unhealthy societies - Wilkinson, RG. J Public Health Med.

[29] McEwen BS (1998). Protective and damaging effects of stress mediators. N Engl J Med.

[30] Shively CA, Clarkson TB (1994). Social status and coronary artery atherosclerosis in female monkeys. Arterioscler Thromb.

[31] Jutz R (2015). The role of income inequality and social policies on income-related health inequalities in Europe. Int J Equity Health.

[32] Chaix B, Rosvall M, Lynch J, Merlo J (2006). Disentangling contextual effects on cause-specific mortality in a longitudinal 23-year follow-up study: impact of population density or socioeconomic environment?. Int J Epidemiol.

[33] Hagstad S, Ekerljung L, Lindberg A, Backman H, Ronmark E, Lundback B (2012). COPD among non-smokers - report from the obstructive lung disease in Northern Sweden (OLIN) studies. Respir Med.

[34] Lindberg A, Bjerg A, Ronmark E, Larsson LG, Lundback B (2006). Prevalence and underdiagnosis of COPD by disease severity and the attributable fraction of smoking report from the obstructive lung disease in northern sweden studies. Respir Med.

[35] International Statistical Classification of Diseases and Related Health Problems 10th revision (ICD-10) WHO version for 2016

[36] Merlo J (2014). Invited commentary: multilevel analysis of individual heterogeneity-a fundamental critique of the current probabilistic risk factor epidemiology. Am J Epidemiol.

[37] Merlo J, Mulinari S (2015). Measures of discriminatory accuracy and categorizations in public health: a response to Allan Krasnik's editorial. Eur J Public Health.

[38] Trachtenberg AJ, Dik N, Chateau D, Katz A (2014). Inequities in ambulatory care and the relationship between socioeconomic status and respiratory hospitalizations: a population-based study of a canadian city. Ann Fam Med.

[39] Gerdtham U-G J (2004). M: absolute income, relative income, income inequality and mortality?. J Hum Resour.

[40] Inghammar M, Engstrom G, Lofdahl CG, Egesten A (2012). Validation of a COPD diagnosis from the Swedish inpatient registry. Scand J Public Health.

[41] Lynch J, Kaplan G, Berkman LF, Kawachi I (2000). Socioeconomic position. Social epidemiology.

[42] Danielsson M, Gilliam H, Hemström Ö: Folkhälsorapport. Kapitel 10. Tobaksvanor och tobaksrelaterade besvär. pp. 297. https://www.socialstyrelsen.se/Lists/Artikelkatalog/Attachments/8495/2009-126-71.pdf: Socialstyrelsen; 2009:297.

[43] Forey BA, Thornton AJ, Lee PN (2011). Systematic review with meta-analysis of the epidemiological evidence relating smoking to COPD, chronic bronchitis and emphysema. BMC Pulm Med.

[44] Miravitlles M, Naberan K, Cantoni J, Azpeitia A (2011). Socioeconomic status and health-related quality of life of patients with chronic obstructive pulmonary disease. Respiration.

[45] Nordahl H (2014). Social inequality in chronic disease outcomes. Dan Med J.

[46] Beckman A, Anell A (2013). Changes in health care utilisation following a reform involving choice and privatisation in Swedish primary care: a five-year follow-up of GP-visits. BMC Health Serv Res.

[47] Burstrom B, Burstrom K, Nilsson G, Tomson G, Whitehead M, Winblad U (2017). Equity aspects of the primary health care choice reform in sweden - a scoping review. Int J Equity Health.

[48] Glymour MM, Rudolph KE (2016). Causal inference challenges in social epidemiology: bias, specificity, and imagination. Soc Sci Med.

[49] Merlo J (2003). Multilevel analytical approaches in social epidemiology: measures of health variation compared with traditional measures of association. J Epidemiol Community Health.

[50] Wemrell M, Mulinari S, Merlo J (2017). Intersectionality and risk for ischemic heart disease in Sweden: categorical and anti-categorical approaches. Soc Sci Med.

